# 2-*tert*-Butyl-6-[(4-chloro-2-nitro­phen­yl)diazen­yl]-4-methylphenol

**DOI:** 10.1107/S1600536809019631

**Published:** 2009-06-06

**Authors:** Hui-Liang Wen, Xiao-Qin Wu, Bo-Wen Lai

**Affiliations:** a State Key Laboratory of Food Science and Technology, Nanchang University, Nanchang 330047, People’s Republic of China; b Department of Chemistry, Nanchang University, Nanchang 330047, People’s Republic of China

## Abstract

In the title compound, C_17_H_18_ClN_3_O_3_, the dihedral angle between the planes of the two benzene rings is 1.03 (7)°. The overall conformation of the mol­ecule is influenced, in part, by electron delocalization and by an intra­molecular bifurcated O—H⋯(O,N) hydrogen bonds. The O atoms of the nitro group, one of which serves as an H bond acceptor, are disordered over two sets of sites with refined occupancies of 0.56 (3) and 0.44 (3).

## Related literature

For benzotriazoles as UV absorbers and their applications in industry, see: Ravichandran *et al.* (2002 ▶). *N*-oxides are a key type inter­mediates in the synthesis of benzotriazoles, see: Wen *et al.* (2006 ▶); Crawford (1999 ▶). For the use of green synthetic methods to obtain inter­mediates, see: Tanaka & Toda (2000 ▶).
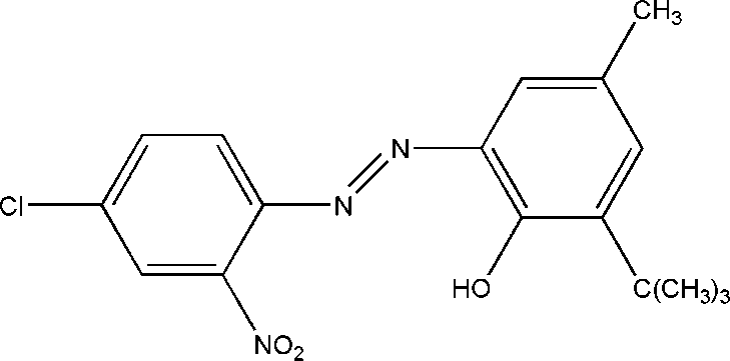


## Experimental

### 

#### Crystal data

C_17_H_18_ClN_3_O_3_
*M* *_r_* = 347.79
Monoclinic, 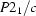

*a* = 14.578 (4) Å
*b* = 7.0616 (19) Å
*c* = 17.043 (5) Å
β = 101.233 (3)°
*V* = 1720.9 (8) Å^3^
*Z* = 4
Mo *K*α radiation
μ = 0.24 mm^−1^
*T* = 296 K
0.31 × 0.18 × 0.16 mm

#### Data collection

Bruker APEXII CCD diffractometer
Absorption correction: multi-scan (*SADABS*; Sheldrick, 1996 ▶) *T*_min_ = 0.930, *T*_max_ = 0.963
14642 measured reflections
3927 independent reflections
2563 reflections with *I* > 2σ(*I*)
*R*_int_ = 0.033

#### Refinement

*R*[*F* ^2^ > 2σ(*F* ^2^)] = 0.044
*wR*(*F* ^2^) = 0.141
*S* = 1.03
3927 reflections
241 parameters
H-atom parameters constrained
Δρ_max_ = 0.22 e Å^−3^
Δρ_min_ = −0.26 e Å^−3^

### 

Data collection: *APEX2* (Bruker, 2006 ▶); cell refinement: *SAINT* (Bruker, 2006 ▶); data reduction: *SAINT*; program(s) used to solve structure: *SHELXS97* (Sheldrick, 2008 ▶); program(s) used to refine structure: *SHELXL97* (Sheldrick, 2008 ▶); molecular graphics: *SHELXTL* (Sheldrick, 2008 ▶); software used to prepare material for publication: *SHELXTL*.

## Supplementary Material

Crystal structure: contains datablocks global, I. DOI: 10.1107/S1600536809019631/lh2824sup1.cif

Structure factors: contains datablocks I. DOI: 10.1107/S1600536809019631/lh2824Isup2.hkl

Additional supplementary materials:  crystallographic information; 3D view; checkCIF report

## Figures and Tables

**Table 1:** Hydrogen-bond geometry (Å, °)

*D*—H⋯*A*	*D*—H	H⋯*A*	*D*⋯*A*	*D*—H⋯*A*
O3—H3⋯O1′	0.82	2.28	2.933 (7)	136
O3—H3⋯O1	0.82	2.50	3.142 (12)	136
O3—H3⋯N2	0.82	1.84	2.553 (2)	145

**Fig. 1.**
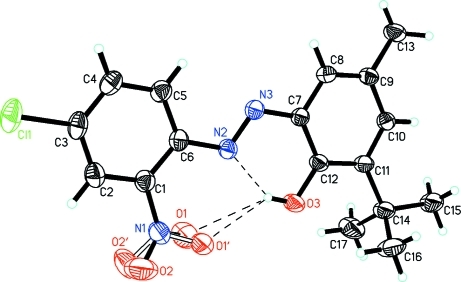
: The molecular structure of the title compound, showing 30% probability displacement ellipsoids. The minor comonent of disorder is shown with open bonds and hydrogen bonds are shown with dashed lines.

## supplementary crystallographic information

### Comment

Benzotriazoles play an important role as a class of UV absorbers and have promising industrial applications (Ravichandran *et al.*, 2002). N-oxides are a key type intermediates in the synthesis of benzotriazoles (Wen **et al.**, 2006; Crawford, 1999) and the title compound is an important intermediate in the synthesis of 2-(2'-Hydroxy-3'-tert-butyl-5'-methylphenyl)-5-chloro benzotriazole (UV 326), a good ultraviolet absorber. Due to the growing awareness of environmental protection, the demand for clean and 'green' (i.e solvent free) chemical syntheses has been growing, so using these synthetic methods to form intermediates have received attention (Tanaka & Toda, 2000). Herein we report a 'green' synthetic method and the crystal structure of the title compound. In the title moleclue (Fig .1) the dihedral angle between the two benzene rings is 1.03 (7)°. The overall conformation of the molecule is influenced, in part, by electron delocalization and by intramolecular O—H···O and O—H···N hydrogen bonds.

### Experimental

The title compound was synthesized *via* the solid phase reaction of 4-chloro-2-nitroaniline and 2-*tert*-butyl-4-substituted phenol at room temperature. After intensive grinding a mixture of 4-chloro-2-nitrobenzenamine 1.72 g (10 mmol), 2-*tert*-butyl-4-methylphenol 1.72 g (10.5 mmol), NaNO_2_ 0.69 g (10 mmol), and KHSO_4_ 1.36 g (10 mmol) in a mortar for 15 min at 293 K, the product was washed with hot water. A few purple crystals suitable for X-ray diffraction analysis were obtained upon recrystallization in ethanol after several days (m. p. 445–446 K), which gave the product in 93% yield and higher than 99% purity (by HPLC).

### Refinement

All H atoms were included in calculated positions with O—H = 0.82Å; *C*—*H*(methyl) = 0.96 Å, C—H(aromatic) = 0.93 Å, and *U*_iso_(H) = 1.5U_eq_(C_methyl_,O) and *U*_iso_(H) = 1.2U_eq_(C) for aromatic H atoms. The O atoms of the nitro group are disordered over two sites with refined occupancies of 0.56 (3) and 0.44 (3).

### Crystal data

**Table:**

C_17_H_18_ClN_3_O_3_	*F*(000) = 728
*M**_r_* = 347.79	*D*_x_ = 1.342 Mg m^−^^3^
Monoclinic, *P*2_1_/*c*	Mo *K*α radiation, λ = 0.71073 Å
Hall symbol: -P 2ybc	Cell parameters from 4044 reflections
*a* = 14.578 (4) Å	θ = 2.6–27.2°
*b* = 7.0616 (19) Å	µ = 0.24 mm^−^^1^
*c* = 17.043 (5) Å	*T* = 296 K
β = 101.233 (3)°	Block, purple
*V* = 1720.9 (8) Å^3^	0.31 × 0.18 × 0.16 mm
*Z* = 4	

### Data collection

**Table:**

Bruker APEXII CCD diffractometer	3927 independent reflections
Radiation source: fine-focus sealed tube	2563 reflections with *I* > 2σ(*I*)
graphite	*R*_int_ = 0.033
φ and ω scans	θ_max_ = 27.5°, θ_min_ = 2.4°
Absorption correction: multi-scan (*SADABS*; Sheldrick, 1996)	*h* = −18→18
*T*_min_ = 0.930, *T*_max_ = 0.963	*k* = −9→8
14642 measured reflections	*l* = −22→22

### Refinement

**Table:**

Refinement on *F*^2^	Primary atom site location: structure-invariant direct methods
Least-squares matrix: full	Secondary atom site location: difference Fourier map
*R*[*F*^2^ > 2σ(*F*^2^)] = 0.044	Hydrogen site location: inferred from neighbouring sites
*wR*(*F*^2^) = 0.141	H-atom parameters constrained
*S* = 1.03	*w* = 1/[σ^2^(*F*_o_^2^) + (0.0591*P*)^2^ + 0.5672*P*] where *P* = (*F*_o_^2^ + 2*F*_c_^2^)/3
3927 reflections	(Δ/σ)_max_ = 0.002
241 parameters	Δρ_max_ = 0.22 e Å^−^^3^
0 restraints	Δρ_min_ = −0.26 e Å^−^^3^

### Special details

**Table:**

Geometry. All e.s.d.'s (except the e.s.d. in the dihedral angle between two l.s. planes) are estimated using the full covariance matrix. The cell e.s.d.'s are taken into account individually in the estimation of e.s.d.'s in distances, angles and torsion angles; correlations between e.s.d.'s in cell parameters are only used when they are defined by crystal symmetry. An approximate (isotropic) treatment of cell e.s.d.'s is used for estimating e.s.d.'s involving l.s. planes.
Refinement. Refinement of *F*^2^ against ALL reflections. The weighted *R*-factor *wR* and goodness of fit *S* are based on *F*^2^, conventional *R*-factors *R* are based on *F*, with *F* set to zero for negative *F*^2^. The threshold expression of *F*^2^ > σ(*F*^2^) is used only for calculating *R*-factors(gt) *etc*. and is not relevant to the choice of reflections for refinement. *R*-factors based on *F*^2^ are statistically about twice as large as those based on *F*, and *R*- factors based on ALL data will be even larger.

### Fractional atomic coordinates and isotropic or equivalent isotropic displacement parameters (Å^2^)

**Table:**

	*x*	*y*	*z*	*U*_iso_*/*U*_eq_	Occ. (<1)
Cl1	1.37753 (4)	0.52156 (11)	0.44085 (5)	0.0871 (3)	
O1	0.9625 (4)	0.559 (3)	0.2905 (6)	0.109 (4)	0.56 (3)
O2	1.0758 (7)	0.659 (2)	0.2334 (6)	0.115 (4)	0.56 (3)
O3	0.81516 (9)	0.7932 (3)	0.36241 (8)	0.0666 (5)	
H3	0.8703	0.7643	0.3663	0.100*	
O1'	0.9670 (8)	0.689 (4)	0.2804 (4)	0.100 (5)	0.44 (3)
O2'	1.0687 (12)	0.545 (5)	0.2372 (10)	0.156 (8)	0.44 (3)
N1	1.04252 (15)	0.6167 (4)	0.29115 (12)	0.0765 (6)	
N2	0.98233 (10)	0.7293 (2)	0.43581 (9)	0.0475 (4)	
N3	0.96277 (10)	0.7767 (2)	0.50347 (9)	0.0468 (4)	
C1	1.10633 (13)	0.6202 (3)	0.36932 (12)	0.0528 (5)	
C2	1.19824 (14)	0.5718 (3)	0.36947 (14)	0.0597 (5)	
H2	1.2171	0.5395	0.3221	0.072*	
C3	1.26084 (14)	0.5723 (3)	0.44035 (15)	0.0599 (6)	
C4	1.23301 (14)	0.6162 (3)	0.51114 (14)	0.0606 (5)	
H4	1.2760	0.6122	0.5592	0.073*	
C5	1.14154 (13)	0.6660 (3)	0.51035 (12)	0.0537 (5)	
H5	1.1233	0.6963	0.5582	0.064*	
C6	1.07582 (12)	0.6716 (3)	0.43917 (11)	0.0468 (4)	
C7	0.87202 (12)	0.8321 (3)	0.50305 (10)	0.0425 (4)	
C8	0.85301 (13)	0.8758 (3)	0.57888 (11)	0.0465 (4)	
H8	0.9010	0.8707	0.6236	0.056*	
C9	0.76528 (13)	0.9255 (3)	0.58763 (10)	0.0473 (4)	
C10	0.69528 (13)	0.9379 (3)	0.51815 (11)	0.0485 (5)	
H10	0.6358	0.9748	0.5242	0.058*	
C11	0.70815 (12)	0.8993 (3)	0.44168 (10)	0.0485 (5)	
C12	0.79931 (12)	0.8412 (3)	0.43353 (10)	0.0465 (4)	
C13	0.74126 (16)	0.9610 (4)	0.66868 (11)	0.0646 (6)	
H13A	0.7066	0.8553	0.6831	0.097*	
H13B	0.7042	1.0738	0.6666	0.097*	
H13C	0.7978	0.9763	0.7078	0.097*	
C14	0.62867 (14)	0.9137 (4)	0.36802 (12)	0.0658 (6)	
C15	0.53856 (16)	0.9904 (5)	0.39088 (14)	0.0868 (9)	
H15A	0.5192	0.9069	0.4290	0.130*	
H15B	0.4901	0.9979	0.3439	0.130*	
H15C	0.5502	1.1142	0.4139	0.130*	
C16	0.65620 (18)	1.0504 (5)	0.30642 (14)	0.0959 (10)	
H16A	0.6645	1.1753	0.3290	0.144*	
H16B	0.6077	1.0528	0.2594	0.144*	
H16C	0.7136	1.0087	0.2924	0.144*	
C17	0.60711 (17)	0.7157 (5)	0.33203 (15)	0.0931 (10)	
H17A	0.6629	0.6623	0.3190	0.140*	
H17B	0.5598	0.7250	0.2844	0.140*	
H17C	0.5852	0.6360	0.3701	0.140*	

### Atomic displacement parameters (Å^2^)

**Table:**

	*U*^11^	*U*^22^	*U*^33^	*U*^12^	*U*^13^	*U*^23^
Cl1	0.0412 (3)	0.1022 (5)	0.1217 (6)	0.0083 (3)	0.0251 (3)	0.0142 (4)
O1	0.052 (2)	0.186 (11)	0.084 (3)	−0.001 (4)	−0.001 (2)	−0.047 (5)
O2	0.105 (4)	0.185 (9)	0.056 (3)	−0.001 (5)	0.016 (3)	0.012 (4)
O3	0.0420 (7)	0.1165 (13)	0.0417 (7)	0.0091 (8)	0.0094 (6)	−0.0118 (8)
O1'	0.070 (4)	0.173 (13)	0.055 (3)	0.043 (5)	0.007 (2)	−0.013 (4)
O2'	0.115 (7)	0.255 (18)	0.091 (7)	0.062 (10)	0.007 (5)	−0.080 (9)
N1	0.0612 (13)	0.1073 (17)	0.0606 (12)	0.0158 (13)	0.0106 (10)	−0.0208 (12)
N2	0.0388 (8)	0.0553 (10)	0.0491 (9)	−0.0003 (7)	0.0102 (7)	−0.0025 (7)
N3	0.0412 (8)	0.0512 (9)	0.0478 (9)	−0.0017 (7)	0.0083 (7)	−0.0007 (7)
C1	0.0465 (11)	0.0550 (12)	0.0571 (12)	0.0003 (9)	0.0106 (9)	−0.0026 (9)
C2	0.0503 (12)	0.0634 (14)	0.0702 (14)	0.0041 (10)	0.0232 (11)	0.0006 (11)
C3	0.0385 (10)	0.0570 (13)	0.0869 (16)	0.0007 (9)	0.0186 (11)	0.0079 (11)
C4	0.0429 (11)	0.0655 (13)	0.0699 (14)	−0.0021 (9)	0.0021 (10)	0.0061 (11)
C5	0.0455 (11)	0.0588 (12)	0.0561 (12)	−0.0017 (9)	0.0084 (9)	0.0023 (9)
C6	0.0394 (9)	0.0460 (10)	0.0554 (11)	−0.0029 (8)	0.0104 (8)	−0.0006 (8)
C7	0.0374 (9)	0.0472 (10)	0.0425 (9)	−0.0019 (7)	0.0072 (7)	−0.0006 (8)
C8	0.0469 (10)	0.0531 (11)	0.0378 (9)	−0.0022 (8)	0.0038 (8)	0.0008 (8)
C9	0.0515 (11)	0.0542 (11)	0.0380 (9)	−0.0012 (9)	0.0131 (8)	0.0004 (8)
C10	0.0418 (10)	0.0617 (12)	0.0441 (10)	0.0039 (8)	0.0139 (8)	0.0015 (9)
C11	0.0402 (10)	0.0668 (13)	0.0384 (9)	−0.0008 (9)	0.0074 (7)	0.0002 (9)
C12	0.0418 (10)	0.0606 (12)	0.0381 (9)	0.0003 (8)	0.0102 (8)	−0.0032 (8)
C13	0.0651 (13)	0.0909 (17)	0.0408 (10)	0.0048 (12)	0.0178 (10)	−0.0003 (11)
C14	0.0399 (11)	0.115 (2)	0.0415 (10)	0.0116 (11)	0.0065 (8)	−0.0021 (11)
C15	0.0470 (12)	0.156 (3)	0.0552 (13)	0.0292 (15)	0.0053 (10)	0.0027 (15)
C16	0.0679 (16)	0.170 (3)	0.0488 (13)	0.0225 (18)	0.0092 (11)	0.0315 (16)
C17	0.0536 (14)	0.154 (3)	0.0663 (15)	−0.0061 (16)	−0.0019 (11)	−0.0380 (17)

### Geometric parameters (Å, °)

**Table:**

Cl1—C3	1.737 (2)	C8—H8	0.9300
O1—N1	1.233 (8)	C9—C10	1.407 (3)
O2—N1	1.217 (8)	C9—C13	1.511 (2)
O3—C12	1.322 (2)	C10—C11	1.379 (2)
O3—H3	0.8200	C10—H10	0.9300
O1'—N1	1.195 (7)	C11—C12	1.423 (2)
O2'—N1	1.179 (10)	C11—C14	1.537 (3)
N1—C1	1.469 (3)	C13—H13A	0.9600
N2—N3	1.285 (2)	C13—H13B	0.9600
N2—C6	1.413 (2)	C13—H13C	0.9600
N3—C7	1.378 (2)	C14—C17	1.534 (4)
C1—C2	1.382 (3)	C14—C16	1.536 (4)
C1—C6	1.397 (3)	C14—C15	1.540 (3)
C2—C3	1.365 (3)	C15—H15A	0.9600
C2—H2	0.9300	C15—H15B	0.9600
C3—C4	1.381 (3)	C15—H15C	0.9600
C4—C5	1.376 (3)	C16—H16A	0.9600
C4—H4	0.9300	C16—H16B	0.9600
C5—C6	1.392 (3)	C16—H16C	0.9600
C5—H5	0.9300	C17—H17A	0.9600
C7—C8	1.408 (2)	C17—H17B	0.9600
C7—C12	1.429 (3)	C17—H17C	0.9600
C8—C9	1.362 (3)		
			
C12—O3—H3	109.5	C9—C10—H10	117.5
O2'—N1—O1'	119.6 (10)	C10—C11—C12	116.78 (16)
O2—N1—O1	126.8 (8)	C10—C11—C14	122.62 (17)
O2'—N1—C1	118.1 (7)	C12—C11—C14	120.59 (16)
O1'—N1—C1	122.2 (5)	O3—C12—C11	119.79 (16)
O2—N1—C1	116.6 (5)	O3—C12—C7	120.99 (16)
O1—N1—C1	116.4 (6)	C11—C12—C7	119.20 (15)
N3—N2—C6	114.81 (15)	C9—C13—H13A	109.5
N2—N3—C7	116.74 (15)	C9—C13—H13B	109.5
C2—C1—C6	122.12 (19)	H13A—C13—H13B	109.5
C2—C1—N1	116.10 (18)	C9—C13—H13C	109.5
C6—C1—N1	121.77 (17)	H13A—C13—H13C	109.5
C3—C2—C1	118.8 (2)	H13B—C13—H13C	109.5
C3—C2—H2	120.6	C17—C14—C16	111.1 (2)
C1—C2—H2	120.6	C17—C14—C11	109.2 (2)
C2—C3—C4	120.94 (19)	C16—C14—C11	110.12 (19)
C2—C3—Cl1	119.29 (17)	C17—C14—C15	107.7 (2)
C4—C3—Cl1	119.75 (18)	C16—C14—C15	107.5 (2)
C5—C4—C3	119.8 (2)	C11—C14—C15	111.17 (17)
C5—C4—H4	120.1	C14—C15—H15A	109.5
C3—C4—H4	120.1	C14—C15—H15B	109.5
C4—C5—C6	121.11 (19)	H15A—C15—H15B	109.5
C4—C5—H5	119.4	C14—C15—H15C	109.5
C6—C5—H5	119.4	H15A—C15—H15C	109.5
C5—C6—C1	117.11 (17)	H15B—C15—H15C	109.5
C5—C6—N2	122.56 (17)	C14—C16—H16A	109.5
C1—C6—N2	120.32 (17)	C14—C16—H16B	109.5
N3—C7—C8	114.70 (16)	H16A—C16—H16B	109.5
N3—C7—C12	124.97 (16)	C14—C16—H16C	109.5
C8—C7—C12	120.30 (16)	H16A—C16—H16C	109.5
C9—C8—C7	120.93 (17)	H16B—C16—H16C	109.5
C9—C8—H8	119.5	C14—C17—H17A	109.5
C7—C8—H8	119.5	C14—C17—H17B	109.5
C8—C9—C10	117.79 (16)	H17A—C17—H17B	109.5
C8—C9—C13	122.19 (17)	C14—C17—H17C	109.5
C10—C9—C13	120.00 (17)	H17A—C17—H17C	109.5
C11—C10—C9	124.95 (17)	H17B—C17—H17C	109.5
C11—C10—H10	117.5		
			
C6—N2—N3—C7	−179.36 (15)	N2—N3—C7—C8	177.70 (16)
O2'—N1—C1—C2	12 (2)	N2—N3—C7—C12	−0.3 (3)
O1'—N1—C1—C2	−165.3 (16)	N3—C7—C8—C9	−177.44 (18)
O2—N1—C1—C2	−32.9 (10)	C12—C7—C8—C9	0.7 (3)
O1—N1—C1—C2	142.5 (11)	C7—C8—C9—C10	−2.1 (3)
O2'—N1—C1—C6	−168 (2)	C7—C8—C9—C13	176.00 (19)
O1'—N1—C1—C6	14.0 (16)	C8—C9—C10—C11	1.5 (3)
O2—N1—C1—C6	146.4 (10)	C13—C9—C10—C11	−176.6 (2)
O1—N1—C1—C6	−38.2 (11)	C9—C10—C11—C12	0.6 (3)
C6—C1—C2—C3	0.7 (3)	C9—C10—C11—C14	179.5 (2)
N1—C1—C2—C3	−179.9 (2)	C10—C11—C12—O3	176.69 (19)
C1—C2—C3—C4	1.4 (3)	C14—C11—C12—O3	−2.2 (3)
C1—C2—C3—Cl1	−177.30 (16)	C10—C11—C12—C7	−2.0 (3)
C2—C3—C4—C5	−2.0 (3)	C14—C11—C12—C7	179.05 (19)
Cl1—C3—C4—C5	176.67 (17)	N3—C7—C12—O3	0.7 (3)
C3—C4—C5—C6	0.5 (3)	C8—C7—C12—O3	−177.22 (18)
C4—C5—C6—C1	1.5 (3)	N3—C7—C12—C11	179.40 (18)
C4—C5—C6—N2	−177.41 (19)	C8—C7—C12—C11	1.5 (3)
C2—C1—C6—C5	−2.1 (3)	C10—C11—C14—C17	−114.1 (2)
N1—C1—C6—C5	178.6 (2)	C12—C11—C14—C17	64.7 (3)
C2—C1—C6—N2	176.80 (19)	C10—C11—C14—C16	123.6 (2)
N1—C1—C6—N2	−2.5 (3)	C12—C11—C14—C16	−57.5 (3)
N3—N2—C6—C5	0.2 (3)	C10—C11—C14—C15	4.6 (3)
N3—N2—C6—C1	−178.68 (17)	C12—C11—C14—C15	−176.6 (2)

### Hydrogen-bond geometry (Å, °)

**Table:**

*D*—H···*A*	*D*—H	H···*A*	*D*···*A*	*D*—H···*A*
O3—H3···O1'	0.82	2.28	2.933 (7)	136
O3—H3···O1	0.82	2.50	3.142 (12)	136
O3—H3···N2	0.82	1.84	2.553 (2)	145
